# Dual-wavelength multifunctional metadevices based on modularization design by using indium-tin-oxide

**DOI:** 10.1038/s41598-018-36595-7

**Published:** 2019-01-23

**Authors:** Jing Luan, Lirong Huang, Yonghong Ling, Wenbing Liu, Chunfa Ba, Shuang Li, Li Min

**Affiliations:** 1 Wuhan National Laboratory for Optoelectronics, School of Optical and Electronic Information, Huazhong University of Science and Technology, 1037 Luoyu Rd, Wuhan, 430074 China; 2 Department of Physics and Electronics, Hunan Institute of Science and Technology, Yueyang, 414000 China

## Abstract

Combining two or several functionalities into a single metadevice is of significant importance and attracts growing interest in recent years. We here introduce the concept of modularization design in dual-wavelength multifunctional metadevice, which is composed of a lower metasurface and an upper metasurface with an indium-tin-oxide (ITO) layer. Benefiting from the fact that ITO holds high infrared (IR) reflection while transparence at visible wavelengths, the metadevice can work in reflection and transmission modes at two very distinct wavelengths, one is 2365 nm in the IR band and the other 650 nm in the visible range. More interestingly and importantly, the two metasurface layers with different functionalities are easy to flexibly integrate into a series of dual-wavelength multifunctional metadevices, with negligible interaction between them and no need of re-designing or re-optimizing their structure parameters. Based on modularization design and functional integration, four kinds of dual-wavelength multifunctional metadevices are demonstrated, which can perform reflective deflection/focusing at 2365 nm and transmissive deflection/focusing at 650 nm. We believe our work may open a straight-forward and flexible way in designing multi-wavelength multifunctional metadevices and photonic integrated devices.

## Introduction

Integrating multiple independent functionalities into one single photonic device is highly desired in photonics integration and has been extensively investigated. Metasurfaces^1–3^, a two-dimensional metamaterial^4,5^, due to their extraordinary light manipulation abilities and advantages in on-chip integration, have attracted significant attention and found applications in beam steering, beam splitting or shaping, holographic imaging, polarization conversion, surface plasmon polariton (SPP) excitation, and so on^6–16^. Although they exhibit various optical manipulation abilities, that most reported metasurfaces are just for a single function. Fortunately, some efforts have been made to overcome this, mobilizing a growing interest in multifunctional metadevices^17–33^, which have one or more metasurface layers and thus are able to carry out two or more functionalities at one or more operation wavelengths. According to their layer structure, multifunctional metadevices can be classified into two types: single-layer type and multilayer type. The first type has only one layer of metasurface^17–21^, whereas the second type employs two or more layers of metasurfaces^22–28^. Most of them perform different functions for different polarization states of the same incoming optical wave^17,18^ or for different operation wavelengths^20,29^.

Multilayer-based multifunctional metadevices have advantage over their single-layer counterparts in the diversity of functionality. However, the functionalities are usually achieved based on the interaction or coupling effect between the constituent upper and lower metasurface layers^26–28^. This hinders independent and flexible design in multilayer metadevices, not only complicating design process but also increasing fabrication difficulty and cost. In addition, many multifunctional devices can only work in pure reflection or transmission mode, not making good use of the full space^18,31^. Moreover, large wavelength contrast-ratio are sometimes highly desired for multi-wavelength metadevices, however, most previously reported metadevices have lower contrast-ratio with operation wavelengths located in the same electromagnetic waveband. For example, the two operation wavelengths of dual-wavelength metadevices are both in the NIR range^20^, THz band^24^, or millimeter regime^26^.

To circumvent these limitations, we here propose a series of dual-wavelength multifunctional metadevices, which can realize multiple functionalities at two very distinct wavelengths respectively, one is 2365 nm in the infrared (IR) band for reflection mode, while the other 650 nm in the visible range for transmission mode. It is composed of a lower metasurface layer and an upper metasurface layer with an indium-tin-oxide (ITO) layer. Benefiting from the unique wavelength-selective reflection and transmission of ITO layer, the two metasurfaces have negligible coupling effect between each other, hence their functionalities can be independently designed and individually optimized at the two operation wavelengths. More interestingly and importantly, employing the concepts of modularization design and functional integration, we can freely and undisturbedly combine the two metasurfaces into a series of dual-wavelength multifunctional metadevices, with little interference between them and without the need to re-design or re-optimize their structural parameters, just like a simple building-block approach. These bring much flexibility and convenience to the design of multifunctional metadevices.

This paper is organized as follows. First, we introduce working principle and device structure; then, we give the modularization design of basic functional modules (blocks); and next we illustrate four examples of functional integration, and present the results and discussion. Finally, a brief discussion is given.

## Working Principle and Device Structure

### Wavelength-selective reflection and transmission of indium-tin-oxide (ITO)

Indium tin oxide (ITO) is the most widely used transparent conducting materials due to its high electrical conductivity and high transmittance in the visible and near-IR range. Consisting of 90%wt (weight percent) indium oxide (In_2_O_3_) and 10%wt tin oxide (SnO_2_)^34,35^, it is also one kind of oxide semiconductors with a wide bandgap over 3.5 eV, and hence visible light cannot excite electron interband transition, therefore it shows optical transparency in the visible and near-IR range. Meanwhile, due to highly doped carriers, ITO has relatively low electric resistivity, which exhibits negative index of refractive in the IR band. Consequently, ITO becomes metal-like and highly reflective in the IR band while transparent to visible light^36,37^.

According to Drude model, the dielectric permittivity of ITO can be written as^38^:

1 $$\varepsilon (\omega )=\varepsilon ^{\prime} +i\varepsilon ^{\prime\prime} ={\varepsilon }_{\infty }-\,\frac{{\omega }_{p}^{2}}{\omega (\omega +i{\rm{\Gamma }})}$$

where *ε*_∞_ = 3.8 is the background permittivity at high frequency, *ω*_p_ = 5.31 × 10^14^ rad/s is plasmonic angular frequency, and *Γ* = 2.7 × 10^13^ rad/s is carrier damping rate.

**Figure 1:**
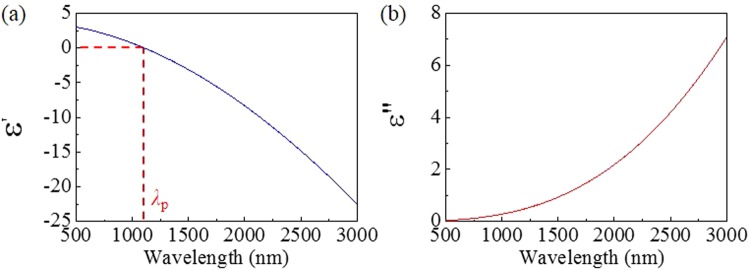
(**a**) Real and (**b**) imaginary parts of dielectric permittivity of ITO films at different wavelengths.

According to formula (1), we plot the real and imaginary parts of *ε*(ω) in Fig. 1. As wavelength increases, the real part of dielectric permittivity *ε*′ decreases from positive to negative value. Here, we define a crossover wavelength *λ*_p_ (where *ε*′ = 0). When wavelength is smaller than *λ*_p_, ITO exhibits dielectric-like property with high transmission. On the contrary, plasmonic property with high reflection is observed in the IR spectral range.

### Device structure and operation principle of dual-wavelength multifunctional metadevice

**Figure 2:**
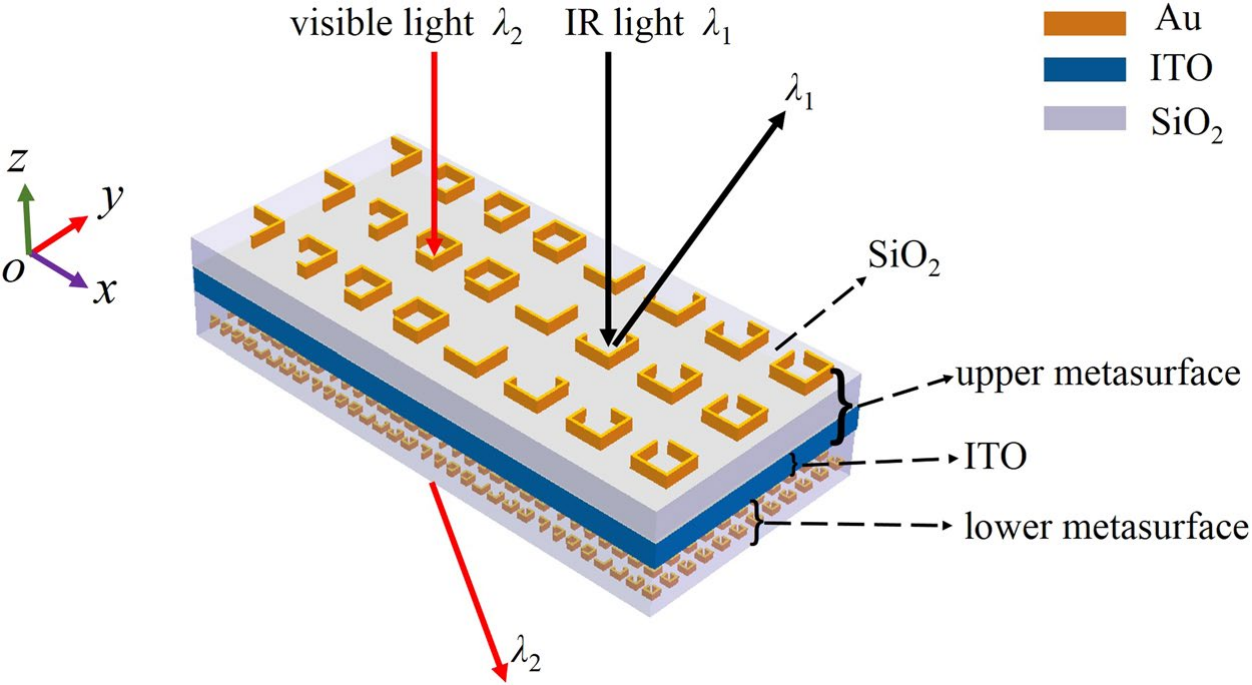
Schematic of the dual-wavelength multifunctional metadevice.

Based on the high IR reflectivity while good visible transparence of ITO material, we propose a novel dual-wavelength multifunctional metadevice, which is composed of an upper metasurface and a lower metasurface, as shown in Fig. 2. The former consists of a gold (Au) antenna array on the top of a silica (SiO_2_) layer and the underneath ITO thin layer, it is designed to offer the IR incident light *λ*_1_ with a desired reflective optical phase profile *φ*_r_(*x*), which determines the behavior of the reflective optical beam. In contrast, the lower metasurface, containing another array of Au antenna units embedded in another SiO_2_ layer, is designed to impart an appropriate transmissive phase profile *φ*_t_(*x*) to the visible light *λ*_2_.

When the IR light *λ*_1_ impinges on the metadevice from the upper metasurface side (i.e., along the negative *z*-axis), the ITO layer behaves like a highly reflective metal ground, the metadevice hence operates in reflection mode and functions like a metal-insulator-metal (MIM) metasurface, and then the IR light *λ*_1_ has little chance to enter the lower metasurface, therefore, it only experiences the reflective phase profile *φ*_r_(*x*) imposed by the upper metasurface. By contrast, when the visible light *λ*_2_ illuminates the metadevice, it can pass through the ITO layer with good transmission and then go into the lower metasurface, experiencing the transmissive phase profile *φ*_t_(*x*) provided by the lower metasurface.

As is well-known, a metasurface carries out a desired functionality when it imposes an appropriate optical phase profile to an optical wave. Therefore, in order to perform different functions at two operation wavelengths, a metasurface should have properly arranged antenna units to provide them with the desired spatial phase distributions.

## Modularization Design of Basic Functional Blocks

Making a good use of high IR reflection and good visible transmission properties of the ITO, we design four basic functional blocks, two of them are related to the upper metasurface, which exhibit reflective deflection and focusing at the IR wavelength *λ*_1_; whereas another two basic functional blocks are associated with the lower metasurface, which perform transmissive deflection and focusing at the visible wavelength *λ*_2_. Based on these basic functional blocks, a series of dual-wavelength multifunctional metadevices can be obtained by combining the two metasurfaces.

### Reflective deflection functional block at *λ*_1_ = 2365 nm(upper)

If we expect anomalous reflection with a predesigned deflection angle by the upper metasurface, it should provide the reflective beam with an optical phase profile *φ*_r1_(*x*), which obeys the generalized Snell’s law^1^:

2 $$\sin ({\theta }_{r})-\,\sin ({\theta }_{i})=\frac{{\lambda }_{1}}{2{n}_{i}\pi }\frac{d{\phi }_{r1}(x)}{dx}$$

where *dφ*_r1_(*x*)*/dx* is the phase gradient along the *x*-axis; *n*_i_ represents the refractive index of incidence media; *θ*_i_ and *θ*_r_ are the angles of incidence and reflection respectively. In our case, *λ*_1_ = 2365 nm, *n*_i_ = 1.

**Figure 3:**
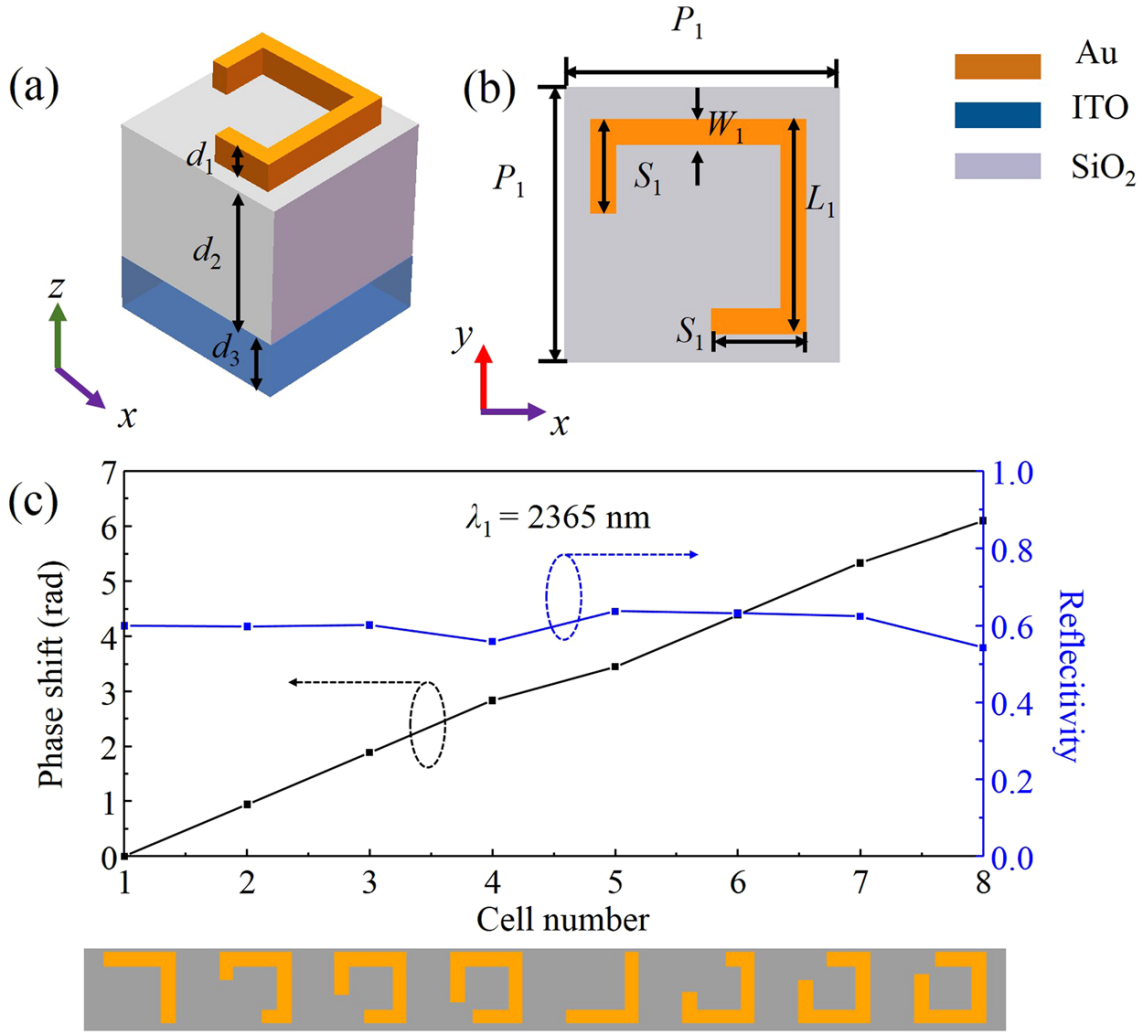
(**a**) Unit cell of the upper metasurface and (**b**) top view; (**c**) cross-polarized reflective phase and reflectivity for the eight Au SRRs (shown at the bottom) at *λ*_1_ = 2365 nm.

To realize this, we design an upper metasurface consisting of an array of Au square split-ring-resonator (SRR) on the top of a SiO_2_ layer and an ITO thin layer, the unit of which is schematically depicted in Fig. 3(a), the thicknesses are *d*_1_ = 150 nm for the SRR, *d*_2_ = 220 nm for the SiO_2_, and *d*_3_ = 160 nm for the ITO, respectively. The other structural parameters are shown in Fig. 3(b), wherein period *P*_1_ = 800 nm, the length *L*_1_ and width *W*_1_ of the SRR are 425 nm and 25 nm, respectively. The arm length *S*_1_ is a variable parameter, which should be optimized to obtain the required reflective phase profile *φ*_r1_(*x*) for the cross-polarized component of the IR light *λ*_1_.

By performing full three-dimensional finite-difference time-domain (FDTD) simulation, we find out the reflective phase and reflectivity dependences on length *S*_1_ and present the results in Fig. 3(c), the bottom panel of which shows a supercell for anomalous reflection, which consists of eight Au SRR unit cells. The period of the supercell is chosen as 6400 nm along the *x*-axis, resulting in a calculated value of 21.6° for *θ*_r_ according to the generalized Snell’s law.

Based on the simulation, we choose the optimized *S*_1_ values of the first four unit cells as 25 nm, 135 nm, 245 nm, 355 nm, respectively. Just like other V- or C-shape antennas in previous literatures^39,40^, additional π phase for the later four unit cells can be obtained by flipping the first four over the *x*-axis. Figure 3(c) indicates that the eight Au antennas contribute uniform cross-polarized reflectivity of nearly 0.6, and other optical energy is mostly absorbed and little is transmitted. Meanwhile, a constant interval of phase difference about π/4 between neighbors can be achieved, thereby providing a total phase shift coverage nearly 2π for the cross-polarized reflected light.

### Reflective focusing functional block at *λ*_1_ = 2365 nm(upper)

If we anticipate the upper metasurface to perform reflective focusing with focal length *f*_r_ at the IR wavelength *λ*_1_ = 2365 nm, it should provide the reflective beam with an optical phase profile *φ*_r2_(*x*), which should be^41,42^:

3 $${\phi }_{r2}(x)=\frac{2\pi }{{\lambda }_{1}}(\sqrt{{x}^{2}+{f}_{r}^{2}}-{f}_{r})$$

in which *f*_r_ is set as 20 μm. Appropriately arranged unit cells can be used to generate the required *φ*_r2_(*x*) to the cross-polarized component of the IR reflected light.

### Transmissive deflection functional block at *λ*_2_ = 650 nm(lower)

Similarly, if one expects anomalous transmission by the lower metasurface at visible wavelength *λ*_2_, the optical phase profile *φ*_t1_(*x*) of the transmitted beam should follow:

4 $${n}_{t}\,\sin \,({\theta }_{t})-{n}_{i}\,\sin \,({\theta }_{i})=\frac{{\lambda }_{2}}{2\pi }\frac{d{\phi }_{t1}(x)}{dx}$$

where *d*φ_t1_(*x*)/*dx* is the transmissive phase gradient along the *x*-axis; *n*_i_ and *n*_t_ represent the refractive indexes of incidence and transmissive media respecticvely; *θ*_i_*, θ*_t_ are the angles of incidence, transmission. In our case, *θ*_i_ = 0, *λ*_2_ = 650 nm.

**Figure 4:**
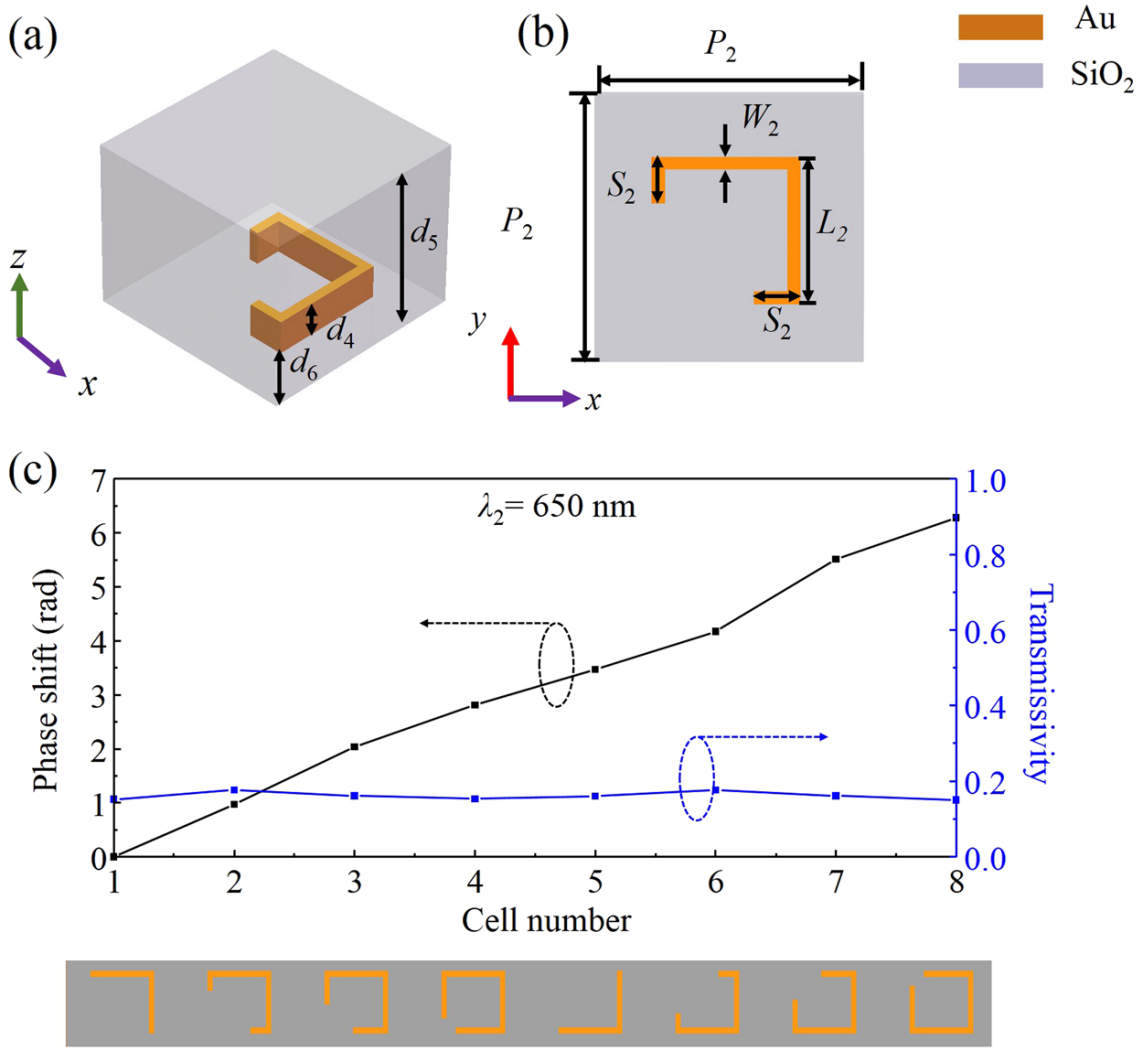
(**a**) Unit cell of the lower metasurface and (**b**) top view; (**c**) Cross-polarized transmissive phase and transmissivity for the eight Au SRRs (shown at the bottom) at *λ*_2_ = 650 nm.

Figure 4(a,b) depict the corresponding structural parameters of the SRR unit cell (period *P*_2_ = 200 nm), which includes another Au square SRR (thickness *d*_4_ = 150 nm) embedded in another SiO_2_ layer (thickness *d*_5_ = 420 nm), the distance *d*_6_ = 50 nm. The length *L*_2_ and width *W*_2_ of the square SRR are 100 nm and 12 nm, respectively. Like *S*_1,_ *S*_2_ is also a variable parameter determining the phase profile *φ*_t1_(*x*) of the cross-polarized component of the transmitted light *λ*_2._

After optimization, we plot the simulated optical phase and transmissivity of the cross-polarized component of the transmitted light *λ*_2_ in Fig. 4(c). The optimized *S*_2_ values of the first four unit cells are 12 nm, 35 nm, 58 nm, 80 nm, respectively; and the later four unit cells can be obtained by flipping the first four over the *x*-axis. Good transmissivity and perfect transmission phase change are seen in the transmission side. These eight unit cells constitute a supercell with a period of 1600 nm along the *x*-axis, resulting in a calculated value of 23.9° for *θ*_t_ according to the generalized Snell’s law.

### Transmissive focusing functional block at *λ*_2_ = 650 nm(lower)

If we hope the lower metasurface to perform transmissive focusing with focal length *f*_t_ for the visible light *λ*_2_, it should provide the transmitted beam with an optical phase profile *φ*_t2_(*x*), which is:

5 $${\phi }_{t2}(x)=\frac{2\pi }{{\lambda }_{2}}(\sqrt{{x}^{2}+{f}_{t}^{2}}-{f}_{t})$$

in which *f*_t_ is set as 20 μm. And we will properly arrange unit cells to induce the required phase distribution *φ*_t2_(*x*) to the cross-polarized component of the transmitted beam.

## Results

After designing the two kinds of reflective functional blocks using the upper metasurface and another two kinds of transmissive functional blocks employing the lower metasurface, we now combine one upper metasurface functional block and one lower metasurface functional block into a metadevice. Just like building block or playing module, four different types of dual-wavelength multifunctional metadevices can be achieved.

### Metadevice 1: Reflective deflection at *λ*_1_ = 2365 nm and transmissive deflection at *λ*_2_ = 650 nm

**Figure 5:**
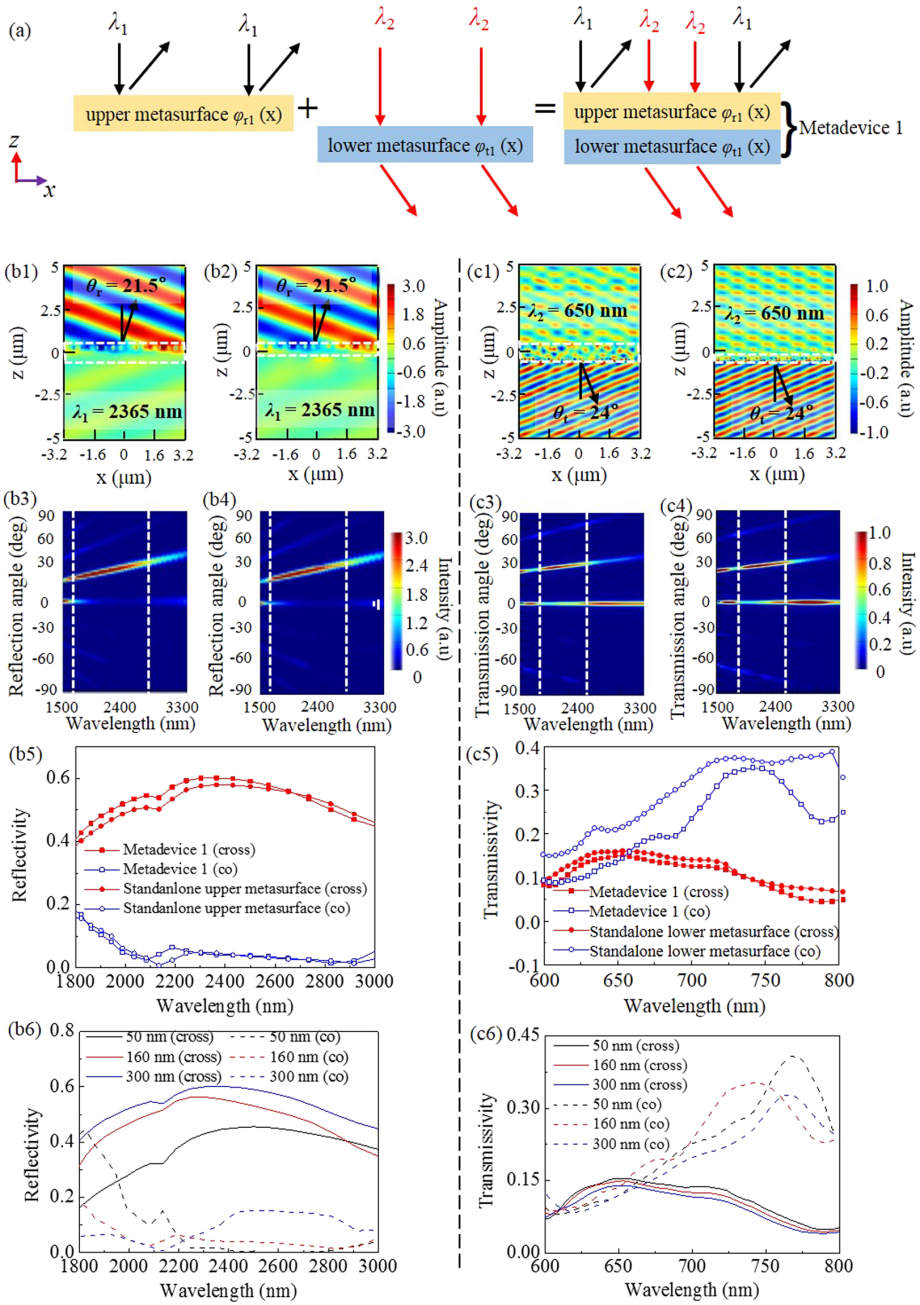
(**a**) Metadevice 1. *Ey* for metadevice 1 (**b1**) and the standalone upper metasurface (**b2**) at 2365 nm. Normalized reflected far-field power intensity for metadevice 1 (**b3**) and the standalone upper metasurface (**b4**). *Ey* for metadevice 1 (**c1**) and the standalone lower metasurface (**c2**) at 650 nm. Normalized transmitted far-field power intensity for metadevice 1 (**c3**) and the standalone lower metasurface (**c4**). Cross-polarized and co-polarized reflectivity spectra in the IR band (**b5**) and transmissivity spectra in the visible band (**c5**). The white dashed rectangles represent the locations of the structures. (**b6**) Cross-polarized and co-polarized reflectivity and (**c6**) transmissivity spectra of metadevice 1 for different ITO thicknesses.

Metadevice 1 functions as a reflective beam deflector at *λ*_1_ = 2365 nm while a transmissive beam deflector at *λ*_2_ = 650 nm, it is the result of combining the upper metasurface functional block with reflective phase profile *φ*_r1_(*x*) and the lower metasurface functional block with transmissive phase profile *φ*_t1_(*x*), as shown in Fig. 5(a).

First, we examine the reflective deflection performance under the normal illumination of *x*-polarized IR light at *λ*_1_ = 2365 nm by mapping the *E*_*y*_ field patterns in the *xz*-plane in Fig. 5(b1). For the purpose of comparison, the corresponding result of the standalone upper metasurface is also presented in Fig. 5(b2), which exhibits almost identical wavefront to that of metadevice 1. It can be seen that when the IR light “hits” the structure, most incident light is reflected to the reflection side at a reflection angle of 21.5°, matching well with the theoretical calculation result of 21.6° according to the generalized Snell’s law given in Eq. (2). As can be seen in Fig. 5(b3,b4), metadevice 1 and the standalone upper metasurface can all work well in abnormal reflection mode in a wide wavelength range. Meanwhile, the cross-polarized and co-polarized reflectivity spectra are plotted in Fig. 5(b5) for metadevice 1 and the standalone upper metasurface, with cross-polarized reflectivity of 0.60 and 0.58 at 2365 nm for metadevice 1 and the standalone upper metasurface, respectively, much larger than the co-polarized reflectivity. Again, these comparisons confirm that the lower metasurface has little interaction or crosstalk with the upper one, and the reason lies in the high reflection of ITO in the IR band, which prevents the IR light from entering the lower metasurface.

After checking the reflection deflection ability of metadevice 1 in the IR waveband, now we evaluate transmissive deflection in the visible band. Under the normal illumination of *x*-polarized light at *λ*_2_ = 650 nm, the *E*_*y*_ field patterns are given in Fig. 5(c1,c2) for metadevice 1 and the standalone lower metasurface. The angles of anomalous transmission are both 24°, which are in good agreement with the theoretically predicted value of 23.9° according to the generalized Snell’s law given in Eq. (4). Comparing with the standalone lower metasurface, the integrated metadevice 1 displays nearly identical transmissive wavefront and slightly reduced transmissivity due to optical loss in the upper metasurface. Figure 5(c1,c2) also indicate the lower metasurface works in both transmission and reflection modes. However, the reflection efficiencies of the anomalous cross-polarized wave at 650 nm for metadevice 1 and the standalone lower metasurface are 0.01, 0.025, respectively, which are much lower than their transmitted counterparts (not shown here). It is worth mentioning that the period of the unit cell of the upper metasurface is 800 nm, which is larger than the operation wavelength 650 nm of the lower metasurface, then the upper metasurface will generate some high order diffractions of co-polarized transmitted waves, which will cause crosstalk between the upper and lower metasurfaces to some degree. In addition, Fig. 5(c3,c4) indicate that metadevice 1 and the standalone lower metasurface work well in abnormal transmission mode with a broad bandwidth. Furthermore, we can see in Fig. 5(c5) that cross-polarized transmissivity of 0.15 at 650 nm for metadevice 1 is a little smaller than 0.16 for the standalone lower metasurface, which is partly due to the above-mentioned high order diffraction effect. The overall comparison results indicate crosstalk or coupling effect between the two metasurfaces is still existent, though at an acceptable level. In addition, Fig. 5(c5) also indicates that the most transmitted wave is co-polarized component in the working band^1^.

It is worth noting that, to eliminate the crosstalk (i.e., coupling effect) between the two metasurface layers, the ITO layer should have a proper thickness. We here investigate the impact of ITO thickness on metadevice 1, and present the simulated cross-polarized and co-polarized reflectivity and transmissivity spectra for different ITO thicknesses. As can be seen from Fig. 5(b6), the reflectivity of cross-polarized light is larger than that of co-polarized light, besides a thicker ITO layer is useful for higher cross-polarized reflectivity in the IR band, whereas a too thin ITO layer (thickness = 50 nm) is not able to isolate the IR light from the lower metasurface, therefore cross-polarized reflectivity in the IR band is remarkably reduced. Meanwhile, we can see from Fig. 5(c6) that most transmitted wave is co-polarized component in the working band^1^. When metadevice 1 has a thinner ITO layer, it can provide the visible light with higher cross-polarized transmissivity. Therefore, we choose the thickness of ITO layer as 160 nm to achieve a better overall performance, and use this value throughout the following three examples.

### Metadevice 2: Reflective focusing at *λ*_1_ = 2365 nm and transmissive focusing at *λ*_2_ = 650 nm

**Figure 6:**
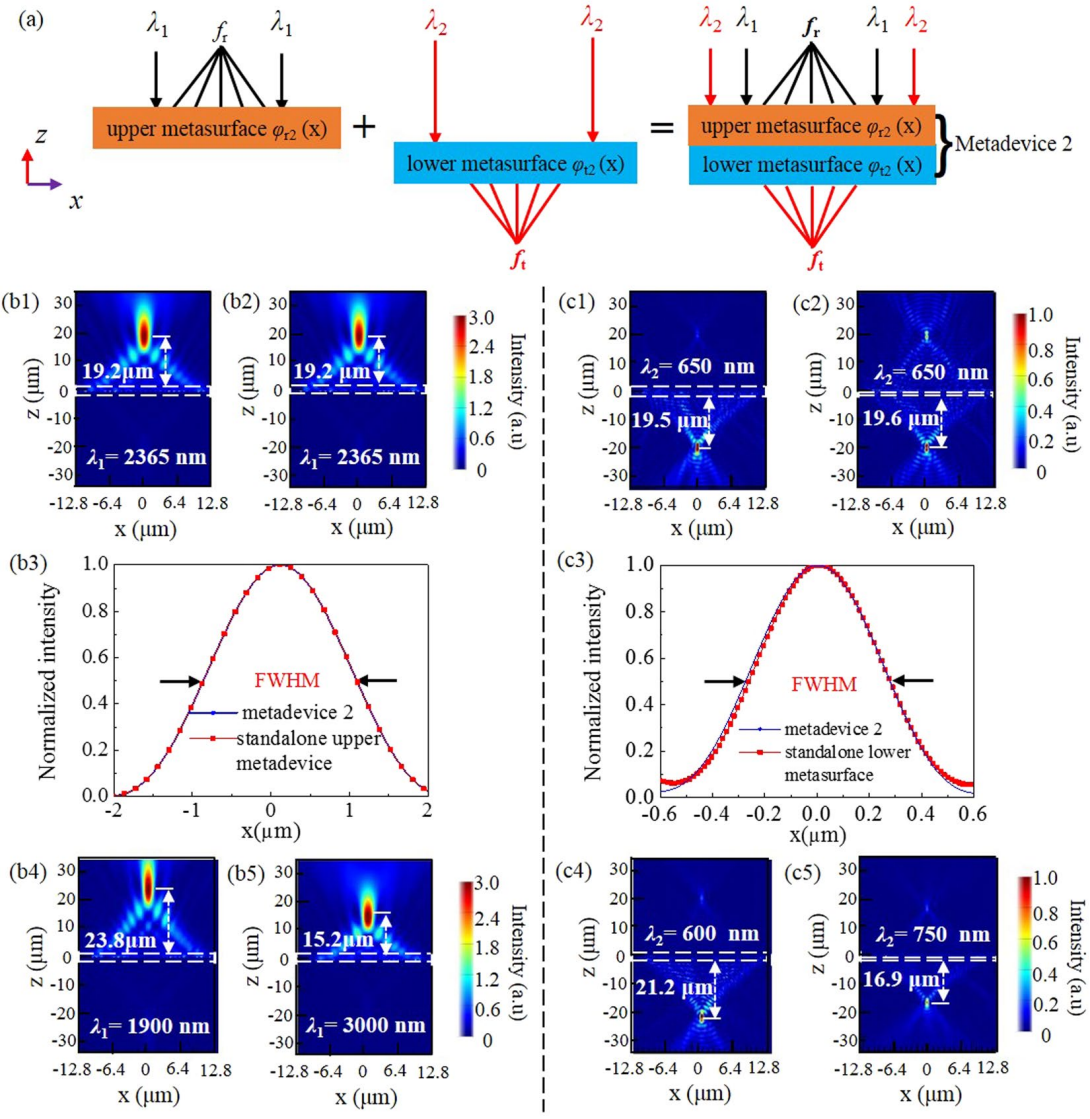
(**a**) Metadevice 2. *|E*_*y*_*|*^2^ for metadevice 2 (**b1**) and the standalone upper metasurface (**b2**) at 2365 nm. *|E*_*y*_*|*^2^ for metadevice 2 (**c1**) and the standalone lower metasurface (**c2**) at 650 nm. Normalized intensity at the horizontal cuts of two reflective focal points (**b3**) and two transmissive focal points (**c3**); *|E*_*y*_*|*^2^ for metadevice 2 at (**b4**) 1900 nm, (**b5**) 3000 nm, (**c4**) 600 nm, (**c5**) 750 nm. The white dashed rectangles represent the locations of the structures.

Metadevice 2 acts as a reflective metalens at *λ*_1_ = 2365 nm while transmissive metalens at *λ*_2_ = 650 nm, it is obtained by combining the upper metasurface functional block with reflective phase profile *φ*_r2_(*x*) and the lower metasurface functional block with transmissive phase profile *φ*_t2_(*x*), as shown in Fig. 6(a).

First, we examine the reflective focusing performance under the normal illumination of *x*-polarized IR light at *λ*_1_ = 2365 nm, the *|E*_y_*|*^2^ distribution in the *xz*-plane is indicated in Fig. 6(b1), and the corresponding result of the standalone upper metasurface is also presented in Fig. 6(b2). Their focus lengths are both found to be 19.2 μm, close to the designed value 20 μm. Enhanced *|E*_y_*|*^2^ field intensity appears around the focal point, while little intensity is seen below the upper metasurface, implying high-efficiency reflective focusing and negligible transmission. For a quantitative analysis of focusing characteristics, we depict the full width at half maximum (FWHM) of electric intensity in Fig. 6(b3). Metadevice 2 and the standalone upper metasurface have the same FWHM of 1941 nm, smaller than the operation wavelength 2365 nm, showing subwavelength focusing ability. It is worth mentioning that the cross-polarized field intensity patterns of metadevice 2 under wavelength 1900 nm and 3000 nm, as shown in Fig. 6(b4,b5), also show good focus phenomena, and this indicates metadevice 2 has a wide operation bandwidth.

In a similar way, we evaluate the transmissive focusing at *λ*_2_ = 650 nm by plotting *|E*_y_*|*^2^ distribution and FWHM in Fig. 6(c1–c3). The focus lengths of metadevice 2 and the standalone lower metasurface are 19.5 μm and 19.6 μm, respectively, both nearly equal the designed value 20 μm. And their FWHM values are found to be 552 nm and 536 nm, respectively. As shown in Fig. 6(c4,c5), at wavelength 600 nm and 750 nm, the cross-polarized field intensity pattern of metadevice 2 also show good transmitted focus effects, indicating a broad operation bandwidth for metadevice 2. As mentioned for metadevice1, the lower metasurface can also generate reflected mode. We here define the reflected focusing efficiency as the ratio of reflected cross-polarized light intensity at the focus point to the incident intensity. The reflection cross-polarized focusing efficiencies for metadevice 2 and the standalone lower metasurface are calculated to be 0.7% and 1.6%, respectively.

### Metadevice 3: Reflective deflection at *λ*_1_ = 2365 nm and transmissive focusing at *λ*_2_ = 650 nm

**Figure 7:**
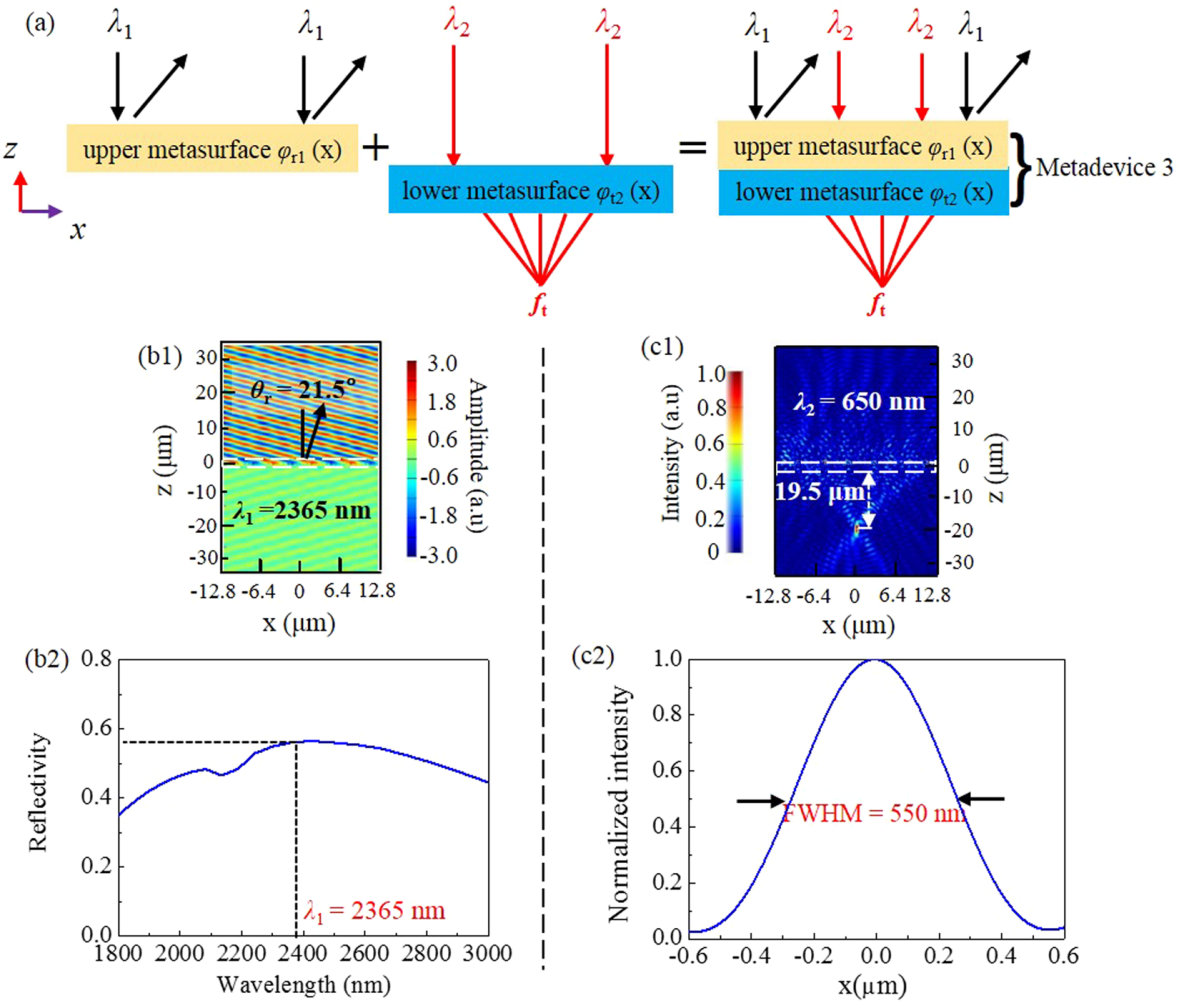
(**a**) Metadevice 3. (**b1**) *E*_*y*_ and (**b2**) cross-polarized reflectivity spectra in the IR band. (**c1**) *|E*_*y*_*|*^2^ and (**c2**) normalized intensity at the horizontal cut of transmissive focal point at 650 nm.

To further discuss the interference between the upper and the lower metasurfaces, we in this subsection propose metadevice 3 shown in Fig. 7(a), which combines the upper metasurface with reflective phase profile *φ*_r1_(*x*) and the lower metasurface with transmissive phase profile *φ*_t2_(*x*). This is to say, it works as a reflective beam deflector at *λ*_1_ = 2365 nm while a transmissive metalens at *λ*_2_ = 650 nm.

From Fig. 7(b1,b2), one can see that, under the normal illumination of IR light *λ*_1_ = 2365 nm, quite nice anomalously reflective wavefront is achieved with a deflective angle 21.5°, matching well with the theoretical value 21.6°. In addition, high-efficiency reflection is seen in a broad wavelength range, and deflecting efficiency at 2365 nm is 0.57, close to 0.58 for the standalone upper metasurface and 0.60 for metadevice 1 example.

In the transmission side, one can see from Fig. 7(c1) that, under normal illumination of visible light *λ*_2_ = 650 nm, transmissive focusing is observed with a focus length of 19.5 μm, which is slightly smaller than 19.6 μm for the standalone lower metasurface discussed in the previous subsection. As shown in Fig. 7(c2), the FWHM value is 550 nm, close to 536 nm for the standalone lower metasurface and 552 nm for metadevice 2. In addition, like metadevice 2, metadevice 3 can achieve a good cross-polarized transmissive focus performance within the same bandwidth (not shown here). It is noted that, compared with Fig. 6(c1), the focus in the upper space in Fig. 7(c1) disappears. We estimate the reason is as follows: the upper and lower metasurfaces in metadevice 2 are both designed to offer parabolic phase distributions. However, for metadevice 3, the antennas in the upper metasurface is periodically arranged to introduce a linear phase distribution, their space arrangement is more regular and ordered than those offering a parabolic phase distribution, hence the upper metasurface has more obvious interference effect on the reflective focusing functionality of the lower metasurface, causing the focus in the upper space point to disappear in Fig. 7 (c1).

### Metadevice 4: Reflective focusing at *λ*_1_ = 2365 nm and transmissive deflection at *λ*_2_ = 650 nm

**Figure 8:**
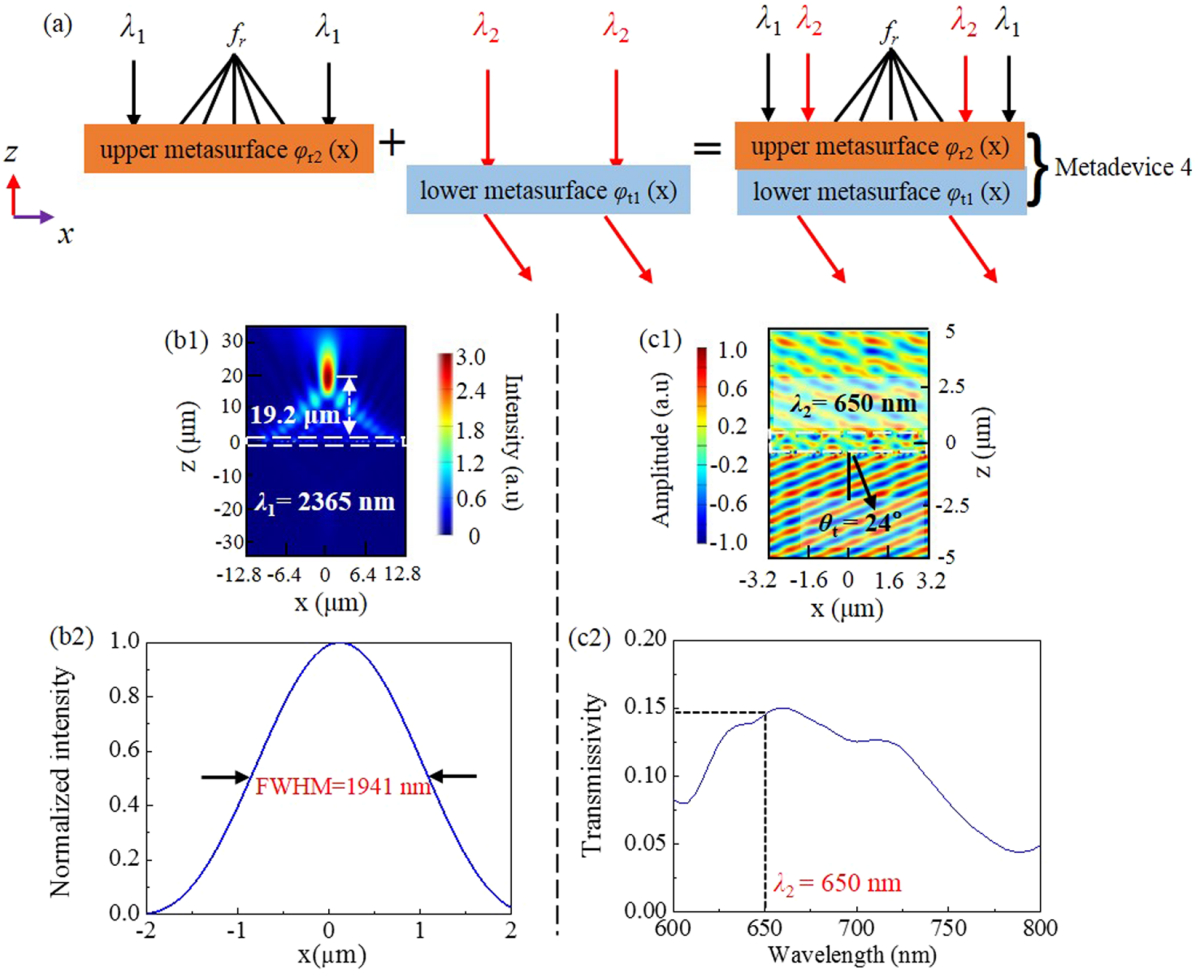
(**a**) Metadevice 4. (**b1**)*|E*_*y*_*|*^2^ and (**b2**) normalized intensity at the horizontal cut of reflective focal point at 2365 nm. (**c1**) *E*_*y*_ and (**c2**) cross-polarized transmissivity spectra in the visible band.

In this example, we design metadevice 4 which combines the upper metasurface with reflective phase profile *φ*_r2_(*x*) and the lower metasurface with transmissive phase profile *φ*_t1_(*x*) shown in Fig. 8(a), it operates as a reflective metalens at *λ*_1_ = 2365 nm while a transmissive beam deflector at *λ*_2_ = 650 nm.

Under the normal illumination of IR light *λ*_1_ = 2365 nm, we find from Fig. 8(b1,b2) a focus length *f*_r_ of 19.2 μm and FWHM value of 1941 nm, consistent to the focus properties of the standalone upper metasurface and metadevice 2 presented in the former subsection. Meanwhile, the working bandwidth keeps close to that of metadevice 2 (not shown here).

As for the transmissive deflection in the visible band, Fig. 8(c1) represents the transmitted *E*_y_ field of metadevice 4 at *λ*_2_ = 650 nm, which achieves anomalously transmissive wavefront with an angle of 24°, close to the theoretical value 23.9°. Figure 8(c2) tells that the transmissive cross-polarized efficiency at 650 nm is 0.145, which is near to 0.16 for the standalone lower metasurface and 0.15 for metadevice 1. In the meantime, it is also found that the cross-polarized anomalous transmission can be realized in a wide wavelength range like metadevice 1 (not shown here).

Obviously, based on the analyses for the above four kinds of metadevices, we can conclude that, because the ITO layer has excellent IR reflection while good visible transmission, when the upper and the low metasurfaces are combined into a metadevice, they can still work well, with little crosstalk or coupling effect between each other, confirming the feasibility of the modularization design and functional integration in the dual-wavelength multifunctional metadevices.

## Discussion

To summarize, choosing ITO layer of appropriate thickness and making a good use of its high IR reflection and good visible transmission properties, we design four kinds of dual-wavelength multifunctional metadevices consisting of a lower metasurface and an upper metasurface with an ITO layer. They have advantages as follows: firstly, they can work at two distinct operation wavelengths and make good use of the full space, with the IR light 2365 nm in the reflection side, whereas the visible light 650 nm in the transmission side; the wavelength contrast-ratio is as high as 3.6. Secondly, based on modularization design and functional integration, the upper metasurface capable of reflective deflection/focusing at 2365 nm and the lower metasurface for transmissive deflection/focusing at 650 nm can be flexibly combined into a series of dual-wavelength multifunctional metadevices, with negligible interaction between the two metasurface layers and no need to re-design or re-optimize their structural parameters, thus significantly simplifying design process and saving fabrication cost. We believe the concepts of modularization design and functional integration open a straight-forward and flexible way in the design of multi-wavelength multifunctional metadevices and photonic integrated devices.

## Methods

Throughout the paper, the numerical simulations are performed by commercial software Lumerical FDTD Solutions. For the simulation of upper and lower unit cells, period boundary conditions along the *x*- and *y*-directions and perfectly matched layer condition along the *z*-direction are applied. For the simulations of the metadevice 1, periodic boundary conditions are set in both *x* and *y* directions for the supercells. For the metadevice 2, 3 and 4, periodic boundary condition is set in *y* direction and perfectly matched layer condition is applied in the *x* direction. The gold is modeled with a lossy Drude dispersion, ${\rm{\varepsilon }}({\rm{\omega }})={\varepsilon }_{\infty }-{{\omega }_{p}}^{2}/\omega (\omega +\gamma )$, where *ε*_∞_ = 7, *ω*_*p*_ = 1.37 × 10^16^ rad/s, γ = 4.08 × 10^13^ rad/s; and the refractive index of SiO_2_ dielectric is set as *n*_*d*_ = 1.5. The mesh of FDTD simulation is set as 5 nm × 5 nm × 5 nm per grid for the whole simulation area.
